# Executive dysfunction in Parkinson’s disease and timing deficits

**DOI:** 10.3389/fnint.2013.00075

**Published:** 2013-10-31

**Authors:** Krystal L. Parker, Dronacharya Lamichhane, Marcelo S. Caetano, Nandakumar S. Narayanan

**Affiliations:** ^1^Department of Neurology, University of Iowa Hospitals and ClinicsIowa City, IA, USA; ^2^Center for Mathematics, Computation and Cognition, Universidade Federal do ABCSanto André, Brazil; ^3^Aging Mind and Brain Initiative, University of Iowa Hospitals and ClinicsIowa City, IA, USA

**Keywords:** temporal processing, executive function, cognitive impairment, Parkinson’s disease, interval timing

## Abstract

Patients with Parkinson’s disease (PD) have deficits in perceptual timing, or the perception and estimation of time. PD patients can also have cognitive symptoms, including deficits in executive functions such as working memory, planning, and visuospatial attention. Here, we discuss how PD-related cognitive symptoms contribute to timing deficits. Timing is influenced by signaling of the neurotransmitter dopamine in the striatum. Timing also involves the frontal cortex, which is dysfunctional in PD. Frontal cortex impairments in PD may influence memory subsystems as well as decision processes during timing tasks. These data suggest that timing may be a type of executive function. As such, timing can be used to study the neural circuitry of cognitive symptoms of PD as they can be studied in animal models. Performance of timing tasks also maybe a useful clinical biomarker of frontal as well as striatal dysfunction in PD.

## INTRODUCTION

Parkinson’s disease (PD) is a neurodegenerative disease where midbrain dopamine neurons inexorably die. Cardinal manifestations of PD include bradykinesia, rigidity, resting tremor, and postural instability. PD patients also have non-motor manifestations such as constipation, salivation, sleep disorders, mood disorders, and cognitive deficits (Chaudhuri and Schapira, 2009; Zesiewicz et al., 2010). Of these, executive dysfunction, one major domain of cognitive deficits in PD (Gotham et al., 1988; Cooper et al., 1991; Aarsland et al., 2010), is associated with considerable morbidity (Williams-Gray et al., 2007) and predicts future mortality (Santangelo et al., 2007; Forsaa et al., 2010).

Executive dysfunction affects roughly 30% of PD patients (Williams-Gray et al., 2009; Aarsland and Kurz, 2010). Deficits in executive tasks may define a disease trajectory, as cognitive symptoms are prognosticators of dementia onset (Mahieux et al., 1998; Levy et al., 2002a,b; Janvin et al., 2005). Such deficits can occur early in the disease (Foltynie et al., 2004; Aarsland et al., 2009) and involve impaired coordination of a range of cognitive processes required to achieve complex, goal-oriented, and novel cognitive operations (Elliott, 2003; Jurado and Rosselli, 2007). Executive processes include working memory, planning, inhibition, attention, and decreased speed of processing (Uc et al., 2005). These processes typically involve the frontal lobe (Fuster, 2008). These impairments are revealed using classic tests of executive function (Van Spaendonck et al., 1996) including verbal fluency, abstract reasoning, picture completion, Stroop performance (Aarsland et al., 2011), and performance on the Tower-of-London task (Foltynie et al., 2004). Executive functions can include inhibitory control (Stuss et al., 2005; Picton et al., 2006) which may be impaired in PD (Wylie et al., 2010; Mirabella et al., 2013). Other executive processes such as action selection can be impaired in PD and correlate with dysfunctional frontostriatal networks (Hughes et al., 2013). Here, we are particularly interested in working memory aspects of executive function, as these appear to be involved in timing (Gibbon et al., 1984) and impaired in PD patients (Malapani et al., 1998).

Importantly, PD-related executive dysfunction is not directly correlated with motor dysfunction (Van Spaendonck et al., 1996), although it has been linked with gait-disturbance (Wylie et al., 2012). Dopaminergic therapy does not reliably improve executive dysfunction in high-functioning (Pascual-Sedano et al., 2008) or moderate PD patients (Morrison et al., 2004) and potentially can have detrimental effects (Cools et al., 2001; Cools and D’Esposito, 2011).

In addition to deficits in executive dysfunction, patients with PD consistently have impaired timing (e.g., Artieda et al., 1992; O’Boyle et al., 1996; Malapani et al., 1998, 2002; Merchant et al., 2008; Jahanshahi et al., 2010; Jones et al., 2011). While some consider timing an executive function (Fuster, 2008), it is not universally considered as such (Elliott, 2003; Jurado and Rosselli, 2007). Executive functions classically involve goal-directed behavior such as planning, flexibility, problem solving, and attentional control (Baddeley and Hitch, 1974; Norman and Shallice, 1986; Lezak et al., 2004) rather than timing. In this review, we discuss evidence that (1) timing tasks involve executive processing in the frontal cortex, and (2) frontal dysfunction may contribute to timing deficits in PD patients.

## PERCEPTUAL TIMING

Timing, i.e., the perception and estimation of time from seconds to minutes, is central in guiding a range of behaviors, from foraging and decision making to goal-directed behavior (Church, 1984). Perceptual timing at this scale can be measured using a variety of interval timing tasks. These tasks require subjects to make responses at precise times indicating their internal and subjective estimates of time. Interval timing is conserved across a wide range of species (Buhusi and Meck, 2005), and is distinct from other measures such as motor timing, implicit timing, ordinal timing, and rhythmic timing.

**FIGURE 1:**
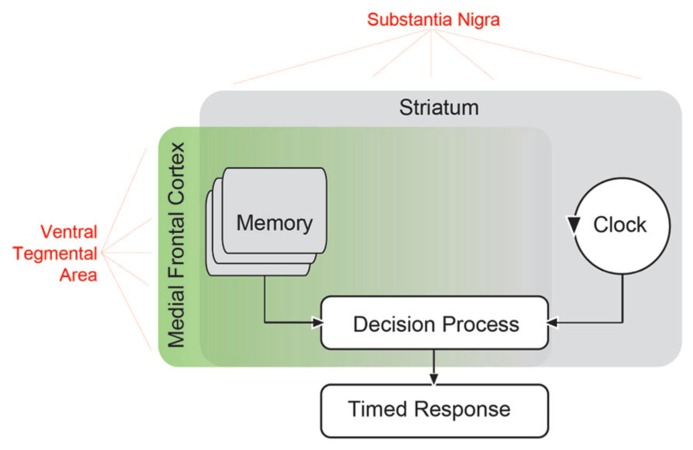
**Scalar timing theory of interval timing based on that proposed by Church (1984).** In this model, an external signal such as a sensory cue starts an internal clock, which compares the passage of time with a criterion stored in working memory. When the criterion approaches the actual clock time, a timed response is initiated. Extensive evidence has suggested that the striatum is involved in all aspects of interval timing (Merchant et al., 2013). Both dorsal and ventral striatum receive dopaminergic input from the substantia nigra via the nigrostriatal pathway, and this pathway can influence timing (Meck, 2006). However, areas in the medial frontal cortex may also be involved (Coull et al., 2011), and likely influence memory as well as decision processes (Mehanna and Jankovic, 2013). These areas receive dopaminergic input (in red) from mesocortical dopamine projections originating from the ventral tegmental area. These mesocortical dopamine projections may contribute to perceptual timing deficits in PD patients.

In order to understand perceptual timing, it is useful to construct a detailed model which accounts both for timing behavior and errors in timing. Understanding the neural basis of timing models can be used to illuminate the mechanism of brain diseases which produce timing errors. One prominent model of timing is the scalar timing theory or scalar expectancy theory, also referred to as the pacemaker–accumulator model of timing (Gibbon et al., 1984). This model assumes the existence of a pacemaker that emits pulses at a certain rate, an accumulator that stores these pulses, and a decision module that constantly compares the accumulated pulses with an example sampled from memory. According to this model (**Figure 1**), a discriminative signal triggers the accumulation of the pulses, which are regularly compared with a randomly sampled number of pulses from the reference memory related to that discriminative signal. When the comparison crosses a threshold, a response is triggered (Church, 1997, 2003). Each component in this model produces variability, and at least of one these must be scalar, producing linearly increasing variance (Gibbon et al., 1984). Of note, there are alternative interval timing theories that, arguing for a lack of biological validity, do not assume the existence of a pacemaker/accumulator-type internal clock (Buhusi and Oprisan, 2013; Laje and Buonomano, 2013); but see Simen et al. (2011) for a clock-like proposal of a neural model to estimate the passage of time). For instance, in a striatal beat–frequency model, striatal medium-spiny neurons serve as timers by detecting and integrating oscillatory states overtime (Matell et al., 2003; Meck et al., 2008; Allman and Meck, 2012). Of these, the scalar timing theory remains the most influential.

A link between interval timing and executive function is that classic tasks loading executive function impair interval timing (Brown, 2006). For instance, remembering items interfered with subjects’ ability to reproduce temporal epochs (Fortin and Rousseau, 1998). When children were asked to both estimate time and perform other executive processes, they were impaired (Rattat, 2010). These type impairments in dual-task experiments suggest that increased dual-task allocation to executive processing resulted in a tradeoff with perceptual timing performance (Brown et al., 2012). Interference seemed to be specifically constrained to the executive process of updating working memory buffers (Ogden et al., 2011). Taken together, these studies provide evidence that interval timing requires executive resources.

Perceptual timing has been reliably linked with dopamine signaling in the striatum (Buhusi and Meck, 2005; Jones and Jahanshahi, 2013). Interval timing can activate medial frontal regions (Hinton et al., 2004; Jahanshahi et al., 2010), including supplementary and pre-supplementary areas (Mita et al., 2009; Coull et al., 2011, 2012; Schneider and Ghose, 2012). Single neurons in medial prefrontal cortex are modulated by time (Niki and Watanabe, 1979; Meck et al., 2008; Genovesio et al., 2009; Narayanan and Laubach, 2009). Rodent studies using pharmacological and optogenetic methods have found that prefrontal D1 dopamine signaling is required for interval timing (Narayanan et al., 2012). A recent temporal discrimination task described that neuronal ensembles in rodent medial frontal cortex were modulated by the passage of time (Kim et al., 2013). These results suggest that interval timing and executive functions share similar neural networks.

Previous reviews have discussed the neuroanatomy of timing in great detail (Matell et al., 2003; Buhusi and Meck, 2005; Meck, 2006; Coull and Nobre, 2008; Coull et al., 2011). A central structure in interval timing is the striatum (Harrington et al., 1998; Jones and Jahanshahi, 2013), likely favoring dorsal striatum (Kurti and Matell, 2011). However, timing can involve diverse and distributed brain networks (Meck et al., 2008; Coull et al., 2011). In **Figure 1**, we have illustrated potential brain networks that underlie timing-related processes suggested by the scalar timing theory. For instance, it is clear that the striatum influences all aspects of perceptual timing, from clock functions to decision and memory mechanisms (Husárová et al., 2011, 2013; Coull et al., 2012; Merchant et al., 2013). While temporal memory is certainly likely to involve frontal networks (Shimamura et al., 1990), recent reviews noted that medial frontal and medial premotor networks were consistently activated by perceptual timing tasks (Coull and Nobre, 2008; Coull et al., 2011). These areas might provide “context” (Merchant et al., 2013) and serve as executive control processes. Many of the frontal areas consistently activated include motor, supplementary motor, and cingulate cortex, which we posit are involved in the response decisions. Systematic recording and disruption experiments from this network in animal models will clarify the role of frontal cortex in memory vs. decision functions during interval timing.

## DOPAMINE AND INTERVAL TIMING

The neurotransmitter dopamine is released from projections originating in the midbrain. Manipulations of dopaminergic signaling profoundly influence interval timing, leading to the hypothesis that dopamine influences internal pacemaker, or “clock,” activity (Maricq and Church, 1983; Buhusi and Meck, 2005, 2009; Lake and Meck, 2013). For instance, amphetamine, which increases concentrations of dopamine at the synaptic cleft (Maricq and Church, 1983; Zetterström et al., 1983) advances the start of responding during interval timing (Taylor et al., 2007), whereas antagonists of D2 type dopamine receptors typically slow timing (Drew et al., 2003; Lake and Meck, 2013). Animal work has indicated that manipulations of both nigrostriatal (Drew et al., 2003, 2007; Meck, 2006) and mesocortical dopamine signaling originating from the ventral tegmental area (Narayanan et al., 2012) can also strongly influence interval timing.

In PD, midbrain projection neurons containing dopamine die (Damier et al., 1999). PD patients are slow to initiate and execute movements (Evarts et al., 1981) and they also have impairments in interval timing. Early studies of PD patients off medication revealed a slowing of timing performance, which correlated with disease severity (Artieda et al., 1992; Pastor et al., 1992), and was consistent with bradykinesia observed with disease progression. Timing deficits were normalized by levodopa, and seem to be constrained to intervals on the scale of seconds (Koch et al., 2008). Notably, these patients had large variability in their estimates of time that was confirmed by a study in PD patients estimating two distinct intervals (Malapani et al., 1998). In this study, PD patients overestimated the short interval and underestimated the long interval; when they estimated the long interval only, they were slower. This effect, dubbed the “migration effect,” has been reliably found in PD patients (Koch et al., 2005). Timing deficits can be rescued by dopaminergic therapy (Malapani et al., 1998), suggesting that beyond clock speed, mnemonic representation for time is impaired in PD and that dopamine influences memory as well as clock subsystems.

Parkinson’s disease patients have timing impairments in related interval timing tasks. For instance, in a duration production and reproduction task with concurrent attentional loads, PD patients were more variable (Perbal et al., 2005). Both levodopa and stimulation of the subthalamic nucleus improved time reproduction (Torta et al., 2010). PD patients can also have deficits in processing temporal information at sub-second time scales (Harrington et al., 1998; Riesen and Schnider, 2001), but there is significant variability in timing at this scale (Merchant et al., 2008; Jones et al., 2011; Claassen et al., 2013). The consistency of PD timing deficits at seconds and inconsistency below milliseconds may be related to the memory and attentional load of longer time intervals (Koch et al., 2008). PD patients consistently tend to have slower, more variable timing over a scale of seconds, which implies that the loss of dopamine influences not only clock deficits in PD patients, but memory and decision processes as well (Mehanna and Jankovic, 2013; **Figure 1**).

Notably, in some studies levodopa normalizes timing (Malapani et al., 1998; Koch et al., 2008) but not other executive functions such as memory, reasoning, or flexible learning (Gotham et al., 1988; Cools et al., 2001; Pascual-Sedano et al., 2008). Dopamine signaling may be complex with non-linearities (Cools and D’Esposito, 2011). Levodopa’s effects may also be influenced by disease severity (Cools et al., 2001). Additionally, PD patients can be heterogeneous (Merchant et al., 2008; Aarsland et al., 2011) with respect to the disease.

Another possibility includes a role for non-dopaminergic signaling in PD-related cognitive dysfunction. Cortical and striatal areas are influenced by other broad neurotransmitter projection systems that are also impaired in PD, such as cholinergic projections originating from basal forebrain (Bigl et al., 1982). This area can be affected in PD patients (Arendt et al., 1983; Whitehouse et al., 1983; Fujita et al., 2006). Furthermore, clinical trials have demonstrated that cholinesterase inhibitor improves cognitive performance for PD patients with mild–moderate dementia (Emre et al., 2004; Poewe et al., 2006). Manipulations of cholinergic signaling (Meck and Church, 1987) and the basal forebrain (Olton et al., 1988) can strongly influence temporal memories. It is unclear if timing deficits are also improved in these patients.

Although PD also involves degeneration of many other brainstem projection nuclei (Scatton et al., 1983; Del Tredici et al., 2002; Jellinger, 2011) such as serotonin and norepinephrine (McCormick et al., 1985), the role of these systems in executive dysfunction and perceptual timing deficits is unclear. However, because levodopa does not reliably treat PD-related cognitive symptoms (Cools et al., 2001, 2002; Morrison et al., 2004; Pascual-Sedano et al., 2008), other non-dopaminergic mechanisms may be important for understanding cognition in PD. Future studies involving detailed correlation of neuropathology with clinical phenotypes as well as specific manipulations of non-dopaminergic systems in animal models will clarify this issue.

## PD AND FRONTAL CORTEX

Deficits in interval timing have been uniformly interpreted as arising from deficits in nigrostriatal dopamine depletion affecting basal ganglia circuitry (Buhusi and Meck, 2005; Coull et al., 2011). However, we note that PD can profoundly influence the function of frontal cortex. The source of cortical dopamine projections in the medial nigral and ventral tegmental midbrain (Williams and Goldman-Rakic, 1998) degenerate in PD (Javoy-Agid and Agid, 1980; Javoy-Agid et al., 1981; Dymecki et al., 1996). These studies suggest that ventral tegmental dopamine neurons degenerate to a lesser extent than the nearby substantia nigra, which primarily projects to the striatum. To date, the functional consequence of ventral tegmental degeneration is unclear (Jellinger, 1999), although there is debate about the involvement of ventral tegmental area in PD (Fearnley and Lees, 1991) likely because there is much overlap between these areas.

Midbrain dopamine neurons encode errors in temporal predictions of reward (Hollerman and Schultz, 1998; Fiorillo et al., 2008; Kobayashi and Schultz, 2008) and can precisely encode temporal information (Lapish et al., 2007). PD involves a dysfunction and loss of these neurons, which could lead to abnormalities in temporal processing in synaptic projection targets in the cortex and striatum. Without this input, these areas can be temporally desynchronized. Depletion of dopamine in healthy volunteers impairs timing (Coull et al., 2012), while amphetamine releases synaptic dopamine and speeds up timing (Taylor et al., 2007). Animal models which record from both dopamine and downstream neurons or dynamically manipulate dopamine neurons while recording in downstream areas will identify the precise role that dopamine release has on temporal encoding in cortex and striatum.

Metabolic imaging demonstrates that PD patients have alterations in prefrontal dopamine signaling (Dubois and Pillon, 1995, 1997) which has been confirmed by positron emission tomography (PET with 18F-DOPA; Rakshi et al., 1999; Kaasinen et al., 2001). These studies have reported that in early PD, prefrontal dopamine uptake has been reported to be increased, presumably due to compensatory mechanisms. Dopamine influences executive function via mesocortical projections originating from the ventral tegmental area (Arnsten and Li, 2005; Cools and D’Esposito, 2011). Prefrontal dopamine release measured by microdialysis is correlated with working memory performance (Phillips et al., 2004). Blocking prefrontal D1 receptors degrades the ability of prefrontal neurons to represent items in working memory (Williams and Goldman-Rakic, 1995; Goldman-Rakic et al., 2004; Wang et al., 2004). In rodent models, prefrontal D1 receptors are associated specifically with interval timing (Narayanan et al., 2012) as well as memory (Seamans et al., 1998; Floresco and Phillips, 2001). In addition, prefrontal dopamine signaling has been linked with a variety of cognitive behaviors, such as reasoning (Takahashi et al., 2012), attentional set-shifting (Floresco et al., 2006), reversal learning (Kehagia et al., 2010), impulsivity (Loos et al., 2010), and decision-making (Floresco, 2013). Dopamine release in prefrontal cortex can modulate network state (Seamans and Yang, 2004). These studies suggest that abnormalities in prefrontal dopamine signaling in PD could impair executive processing (Miller and Cohen, 2001).

Cognitive symptoms of PD appear can be linked with dysfunction in prefrontal networks. Deactivation in medial prefrontal cortex is associated with cognitive dysfunction in PD in metabolic imaging studies (Huang et al., 2007). Brain imaging studies in PD patients have found aberrant prefrontal networks. For example, controls activated medial frontal networks more reliably than PD patients during a task of random numbers generation (Dirnberger et al., 2005). Levodopa restored prefrontal blood flow as measured by PET during performance on a Tower-of-London task (Cools et al., 2002). Decreased performance on attentional set-shifting by PD patients is correlated with less prefrontal metabolic activity (Sawada et al., 2012). This line of research suggests that there is executive dysfunction in PD that may involve the frontal cortex during timing tasks.

Several studies directly engage this question (**Table 1**). In a study of paced-finger tapping, less medial premotor as well as sensorimotor and cerebellar activity was observed in PD patients relative to controls (Elsinger et al., 2003). While on and off levodopa, Harrington et al. (2011) asked PD patients to perform a perceptual timing task, comparing two intervals of time while collecting functional magnetic resonance images (fMRI). This study found temporal impairments throughout frontostriatal and cerebellar networks, and found abnormal activations in medial frontal and parietal areas, which are typically associated with executive processes such as working memory. Activity in this network was abnormal while PD patients encoded time and absent during the decision phase; furthermore, in medial frontal areas such as the cingulate cortex, different temporal profiles were observed in concert with temporal alterations in the striatum. A study modeling effective connectivity found that in controls, medial supplementary motor areas had excitatory coupling with subcortical areas, while PD patients did not (Husárová et al., 2013). Some studies find no changes in this circuit in PD patients during timing tasks (Cerasa et al., 2006; Praamstra and Pope, 2007); of note, this may be related to both disease and pathological heterogeneities in PD patients (Merchant et al., 2008; Aarsland et al., 2011). Nonetheless, PD patients with timing-related dysfunction have abnormalities in medial frontal areas (including supplementary motor areas) in addition to the basal ganglia and cerebellum.

**Table 1:** Studies that examine the neuroanatomical basis of timing deficits and PD.

Study	Task	Subjects	Hypoactive areas in PD patients
Harrington etal. (2011)	Time perception task	Controls PD patients on/and off	Middle frontal cortex/parietal cortex – temporal encoding striatal dysfunction – time keeping
Jahanshahi etal. (2010)	Finger tapping	Controls PD patients on/and off	No activation in medial frontal cortex, cingulate, hippocampus, accumbens during timing
Elsinger etal. (2003)	Finger tapping	Controls PD patients on/and off	Decreased activation in medial premotor cortex, sensorimotor cortex, and cerebellum
Cerasa etal. (2006)	Internally timed movements	Controls PD patients on/and off	Similar supplementary motor and subcortical areas in PD patients OFFand controls
Praamstra and Pope (2007)	Timed choice reaction time task	Controls vs. PD patients	Abnormal beta/alpha activity related to temporal preparation
Husárová etal. (2013)	Motor timing task	Controls vs. PD patients off	Similar area: supplementary motor, basal ganglia, cerebellum, putamen. PD patients had an inhibitory SMA-Cb connection.
Husárová etal. (2011)	Motor timing	Contols vs. PD patients off	Did not include cortical cuts; less basal ganglia/cerebellum in PD patients.

These data are supported with neurophysiological studies from animal models. For instance, in a temporal judgment task, primate prefrontal neurons predominantly encoded temporal duration (Genovesio et al., 2009). These same neurons were robustly modulated during the response window when a judgment was made. Similar patterns have also been observed in rodent frontal cortex (Narayanan and Laubach, 2006, 2009; Kim et al., 2013), and suggest that medial frontal cortex plays an essential role in encoding and judging temporal information.

Notably, the frontal cortex and striatum function together as a circuit (Alexander et al., 1986), and several of the preceding studies involved PD-related abnormalities in the striatum as well as the frontal cortex (Elsinger et al., 2003; Harrington et al., 2011; Husárová et al., 2011) which could be modulated by dopamine (Jahanshahi et al., 2010). Effective connectivity analyses suggest that striatal and cortical areas work together to guide behavior during timing tasks (Husárová et al., 2013). However, investigating the precise mechanistic relationships among areas in the frontal cortex and striatum would require recording from both areas simultaneously or inactivating one area and examining neural activity in the other. Intraoperative recording during deep-brain stimulation surgery (Sheth et al., 2012) or recording from animal models (Narayanan and Laubach, 2006) could investigate this question in detail.

Several investigators have suggested that timing deficits in PD may involve cognitive processing well beyond motor timing such as memory (Malapani et al., 1998; Koch et al., 2008). Imaging studies have captured deficits in control processing originating from medial regions of the frontal cortex (Jahanshahi et al., 2010; Harrington et al., 2011). These data suggest that interval timing and executive functions share resources in the medial frontal cortex, and that dysfunctional timing in Parkinson’s patients may involve frontal as well striatal circuitry.

There are two clear implications supporting this idea. First, interval timing may be a useful clinical tool to assess the integrity of the frontostriatal system (Wild-Wall et al., 2008). One could imagine a specific interval timing paradigm to assess perceptual timing with a range of intervals spanning milliseconds to several seconds targeted at assaying both motor timing and higher order processing. As such, this task could provide early detection of timing deficits. Such a test would be simple, readily added to a neuropsychological battery or even administered remotely (Sternberg et al., 2013) to assist in diagnosis of disease, screen for executive dysfunction, track disease progression, and/or response to therapy.

Secondly, perceptual timing tasks can be readily trained in animals (Coull et al., 2011) such as rodents (Balci et al., 2008) or pigeons (Ludvig et al., 2011). This enables detailed, mechanistic investigation of neural circuits underlying the temporal organization of motivated behavior with tools such as transgenic mice (Drew et al., 2007), optogenetics (Narayanan et al., 2012), and neuronal ensemble recording (Matell et al., 2003; Kim et al., 2013). These circuits can be investigated in detail to understand how frontostriatal circuits are involved in perceptual timing.

In summary, we have suggested that perceptual timing is a type of executive function. Considering it as such implies that testing perceptual timing is a useful way of testing cognitive function in PD patients. Detailed mechanistic understanding of the neural circuits involved in perceptual timing in PD could lead to a greater understanding of the cognitive symptoms of PD and to targeted therapies for this difficult clinical problem.

## Conflict of Interest Statement

The authors declare that the research was conducted in the absence of any commercial or financial relationships that could be construed as a potential conflict of interest.
